# Supplementary material for: Hyperspectral oblique plane microscopy enables spontaneous, label-free imaging of biological dynamic processes in live animals
Source: Proc Natl Acad Sci U S A. 2024 Oct 14;121(43):e2404232121. doi: 10.1073/pnas.2404232121 (PMC11513980; doi:10.1073/pnas.2404232121)
Supplement: Supplementary file 1 — Appendix 01 (PDF) [file pnas.2404232121.sapp.pdf]

Supporting Information for

Hyperspectral Oblique Plane Microscopy Enables Spontaneous, Label-Free Imaging of Biological Dynamic Processes in Live Animals

Ke Guo, Konstantinos Kalyviotis, Periklis Pantazis and Christopher J Rowlands

Christopher J Rowlands.
E-mail: c.rowlands@imperial.ac.uk

This PDF file includes:

Supporting text
Figs. S1 to S7
Table S1
Legends for Movies S1 to S2
SI References

Other supporting materials for this manuscript include the following:

Movies S1 to S2

Supporting Information Text

System design

Water chamber fabrication. In any OPM design, O3 must be tilted with respect to O2, which creates obvious difficulties in capturing the rays propagating at high angles to the optical axis of O3. Traditionally this has been achieved with a high-NA-air-immersion objective (which cannot achieve the NA necessary to capture all the rays emitted by O2), a high-NA water immersion objective with a custom-made water chamber (1) or more recently a dedicated solid-immersion zero-working-distance OPM objective(2, 3). Due to the cost and inflexibility of zero-working-distance lenses, we use a water chamber solution. An existing water-immersion objective (Nikon MRD77220, 20×, 1.1NA) was fitted with a custom-machined water chamber (see Fig. S1(A),(B)) which sat over the lens, and could be positioned accurately using a differential micrometer. O-ring seals ensured the water chamber did not leak, and the outermost optical surfaces consisted of glass coverslips cut to shape and glued onto the chamber housing. A separate mounting clamp held the water chamber tightly to the objective body. A translation stage attached between the clamp and the water chamber was used to adjust the position of O3 so that it focused on the outer surface of the coverslip to minimise spherical aberration.

Grating insert fabrication. λ-OPM is designed to rapidly and easily convert between a conventional OPM design and the hyperspectral imaging configuration. A custom insert for a commercial kinematic fluorescence filter cube (ThorLabs DFM1/M magnetic filter cube) was created by modifying the existing DFM1T1 cube insert. One half of the insert was replacing with a custom-machined kinematic grating mount, and the existing half was milled to make room (see Fig. S1(C,D)). The insert held a grating oriented such that the zero order reflection passes outside the system aperture. The first order diffraction propagates along the optical axis, through the tube lens and images onto the camera. The three-point kinematic adjusters and retaining spring allow for small change of the grating tilt angle and therefore the spectral range detected by the camera.

A separate filter cube, containing a 532 nm dichroic mirror (Semrock DI03-R532-T1-25X36) and two band-pass filters (Semrock FF01-515/30-25 and FF01-582/64-25), could be used instead of the grating cube, allowing easy conversion between the hyperspectral imaging mode and conventional red and green filter channels.

Laser beam profile

The laser beam profile was characterized by taking the fluorescence image of an aqueous solution of quantum dots (Sigma-Aldrich CdSeS/ZnS alloyed 665 nm, diluted) using Camera 1, assuming the laser beam to be circularly symmetric. Fig. S2 shows an example beam profile. After binning the image by 10 in the horizontal direction, we calculate the FWHM of each vertical cut, which is 10 µm in the centre and less than 18 µm on the left and right side of the image. This estimate of the spatial resolution was also used to estimate the spectral resolution. Spectral components dispersed by the grating are focused onto the camera plane. Those spaced one FWHM apart would be resolved at approximately the diffraction limit. Trigonometry can be used to estimate this angle θ, as the grating is located at the back focal plane of the tube lens that forms an image on the camera. This means spot separation d_{ss} is approximated by $d_{ss} \approx \sin\theta \cdot f_{TL}$, where f_{TL} is the focal length of the tube lens and the small angle approximation means the hypotenuse can be substituted for the adjacent. Substituting into the grating equation $d \cdot \sin\theta = m\lambda$ (where d is the grating period, m is an integer and λ is the wavelength) yields $d \cdot \frac{d_{ss}}{f_{TL}} = m\delta\lambda$, where $\delta\lambda$ is the estimated spectral resolution. Rearranging for $\delta\lambda$ gives $\delta\lambda = \frac{d}{m} \cdot \frac{d_{ss}}{f_{TL}} = d_{ss}\frac{d}{f_{TL}}$, when m is taken to be 1. To convert from bandwidths in wavelength to bandwidths in wavenumbers, we use the following:

$$\delta\lambda_w = (\frac{10^7}{\lambda_{laser}} - \alpha) - \frac{10^7}{\frac{10^7}{(\frac{10^7}{\lambda_{laser}} - \alpha)} + \delta\lambda}$$

where $\delta\lambda_w$ is the bandwidth in cm^{-1}, $\delta\lambda$ is in units of nm, λ_{laser} is the laser wavelength in nm and α is the Raman shift in cm^{-1}.

To confirm the spectral resolution, we measured the Raman spectrum of a drop of low refractive index UV adhesive (NOA 1348, n=1.348, Norland Products) as shown in Fig. S2 (c-d), without deconvolution. Two peaks with 100 cm^{-1} separation near 1600 cm^{-1} were well-resolved in the centre of the FOV (blue) but not on the two sides (red and green), indicating a change of resolution from about 100 cm^{-1} to larger values. The peaks near 3000 cm^{-1} are slightly broader than the expected resolution (88 to 160 cm^{-1}) likely due to overlap of two or multiple peaks. The image was taken with a dielectric mirror (Thorlabs BB2-E02) replaced by an unprotected gold mirror (Thorlabs PF20-03-M03) to eliminate the artefact in the middle of the image described in the following section.

Background scattering

The optics near the intermediate image plane introduced some strong fluorescence and scattering spots in the hyperspectral image frame. While these could be eliminated by subtracting a blank image, imprecise subtraction (due to sample effects and laser fluctuations) and photon shot noise occasionally resulted in artefacts (see Fig. S3) or spurious intensity fluctuation at the corresponding x position in the final map, e.g. in Fig. 2(a).

Point spread function (PSF)

The PSF was measured from the images of 210 nm fluorescent beads (Tetraspeck microspheres, 0.2 µm blue/green/orange/dark red) embedded in agarose. To prepare the sample, the bead suspension was diluted in water by 20 times, mixed with 2 % Agarose solution (warm), and finally on a 35 mm glass-bottomed dish to form a pad. The sample was excited using a blue laser (Coherent Obis 488nm LX 150mW) with Galvo y dithered to form a lightsheet. The beamsplitter and filter set was replaced by a beamsplitter and filter set for matching wavelengths (FF497-Di01 and FF01-565/133-25). Images were taken at a few Galvo x settings with 800 nm steps to form a 3D image (after correcting for the skew). To obtain the FWHM of the PSF, the 3D image of each bead was fit individually by a 3D Gaussian function using least square method. The FWHMs of each bead image in the x,y and z directions were calculated from the fitting parameters. Fig. S4(a) shows the distribution of the 3 FWHMs of each bead image as a function of the position in the field of view.

The PSFs in the centre of the field of view (Fig. S4(c)) are similar to Gaussian functions with FWHMs of around 650 nm along the focal plane of O1 and 3400 nm along the optical axis. The FWHMs significantly increase near the edge of the field of view with aberration, as shown in Fig. S4(b,d).

Fluorescence subtraction from spectra data

Autofluorescence signals from zebrafish embryos significantly reduces the contrast of Raman peaks. For better contrast, a broadband spectrum was estimated from each measured spectrum as the autofluorescence spectrum and subtracted.

The autofluorescence spectrum was estimated iteratively as follows: in each iteration, we first smoothed the data (MATLAB `smoothdata`) to obtain a broadband spectrum which was then compared with the original spectrum. For each point where the broadband spectrum was higher than the original spectrum, the intensity was lowered to the original value. The resulting spectrum was again smoothed. After 10 iterations, a broadband spectrum was obtained which approximately follows the valleys of the original spectrum. This spectrum was then used to estimate the autofluorescence and subtracted. Fig. S6(a) shows a comparison between an original spectrum, estimated fluorescence signal and the final spectrum.

This method overestimates the fluorescence around 3000 cm^{-1}, particularly reducing the intensity of the water peak from the heart measurements. Therefore, for the heart measurements, an extra step was added to the iteration. Namely, the estimated fluorescence signal was flattened from 2760 cm^{-1}, as shown in Fig. S6(b).

Transmission

Theoretical system throughput from sample to detector is calculated by multiplying the manufacturer's transmission specifications for each component in the system (see Table S1; an equivalent line-scanning confocal Raman system is also provided for comparison, consisting of, in order from sample to detector: an objective, turning mirror, dichroic, filter, telecentric tube lens, slit, telecentric tube lens, grating and tube lens. The slit is assumed to have a transmission of 100%). We use a reference wavelength of 660 nm (i.e. the laser wavelength) for simplicity for the forward transmission. Experimental confirmation was achieved by measuring the ratio of the power just after the dichroic, and at the sample to get the forward transmission (47%). The backward measurement was conducted by guiding a 685 nm ∼4 mm diameter laser beam from the dichroic through O2, O3, and reflected off the grating. It was measured to be 46%, for a total transmission of 21.6%, not including the dichroic, filter, or final TTL200 tube lens.

Part	Transmission (%)	Number in OPM	OPM transmission (%)	Number in confocal	Confocal transmission (%)
TTL200-A	97.2	1	97.2	1	97.2
TTL200MP	93.6	4	76.6	2	87.5
Nikon MRD77220 *	80	2	64	1	80
Olympus UCPLFLN20X *	86	1	86		
AC508-750-A	99.8	1	99.8		
Grating *	70	1	70	1	70
Dynaxis 3L	95.7	1	95.7		
Di03-R660	99.0	1	99.0	1	99.0
LP02-664RU	99.5	1	99.5	1	99.5
Right-angle mirror	95.7	1	95.7	1	95.7
BBE2-E02	99.6	4	98.3		
Coverslip **	93.5	1	93.5		
**Instrument transmission**			**23.7**		**44.9**

Table S1. Theoretical transmission of λ-OPM, as well as a hypothetical equivalent confocal line-scanning Raman microscope. * Values estimated from manufacturer transmission curve plots. ** Values estimated from the Fresnel equations for unpolarized light going from air to glass, and from glass to water, at 45°.

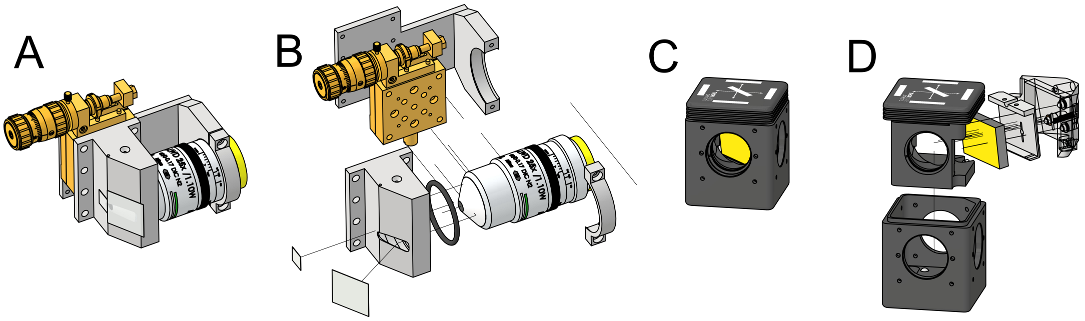

Fig. S1. Custom parts. (A) Assembled water chamber assembled onto a microscope objective. The focal position of the coverslip can be tuned using a differential micrometer. (B) Exploded diagram of (A). (C) Modified fluorescence filter cube containing a grating mounted in a custom kinematic mount. (D) Exploded version of (C), highlighting the custom-machined modified insert, grating mount, three-point kinematic adjusters and retaining spring.

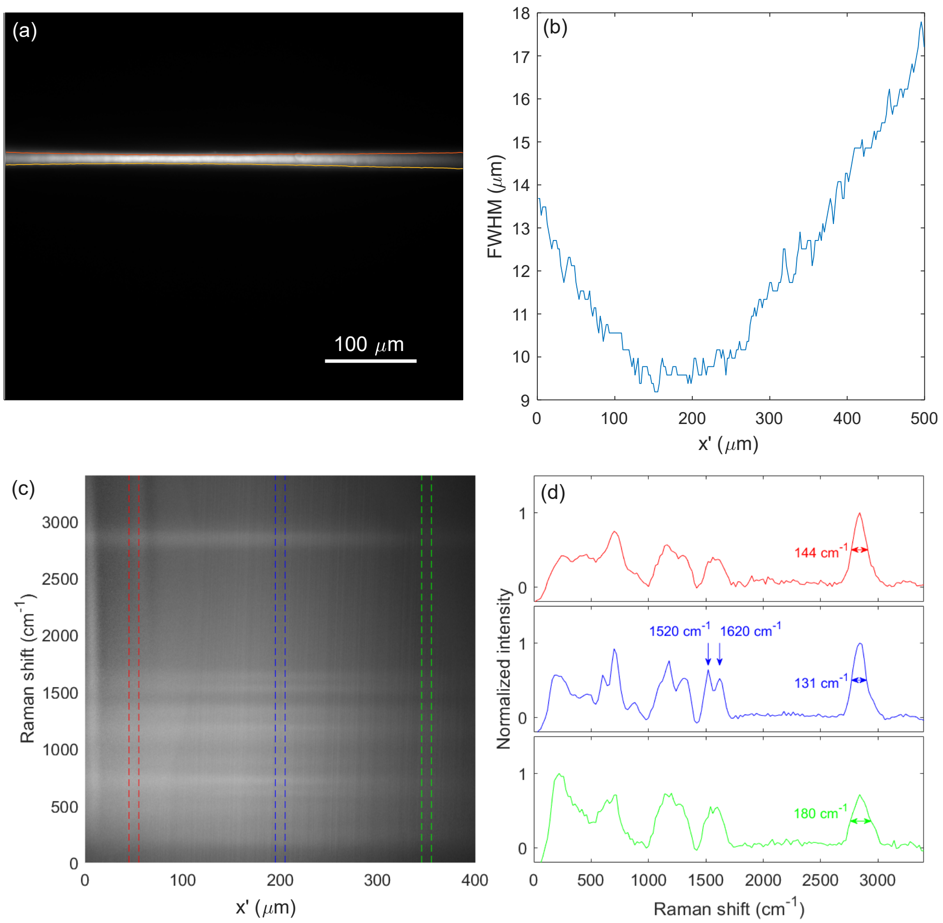

Fig. S2. Laser beam profile and spectral resolution. (a) A fluorescent image of the laser beam measured from an aqueous solution of quantum dots. The red and yellow lines indicate the positions of the half maximum. (b) FWHM of the laser beam as a function of the horizontal position.(c) A hyperspectral Raman image of a drop of low refractive index UV adhesive (NOA 1348, n=1.348). (d) Three spectra obtained from different positions of (c) as indicated by the dashed lines with matching colours. The autofluorescence was estimated and subtracted but no deconvolution was applied. Two peaks with 100 cm^{-1} separation near 1600 cm^{-1} were well resolved in the centre of the FOV (blue) but not on the two sides (red and green), indicating a change of resolution from below 100 cm^{-1} to larger values. The peaks near 3000 cm^{-1} are slightly broader than the expected resolution (80 to 160 cm^{-1}) likely due to overlap of two or multiple peaks.

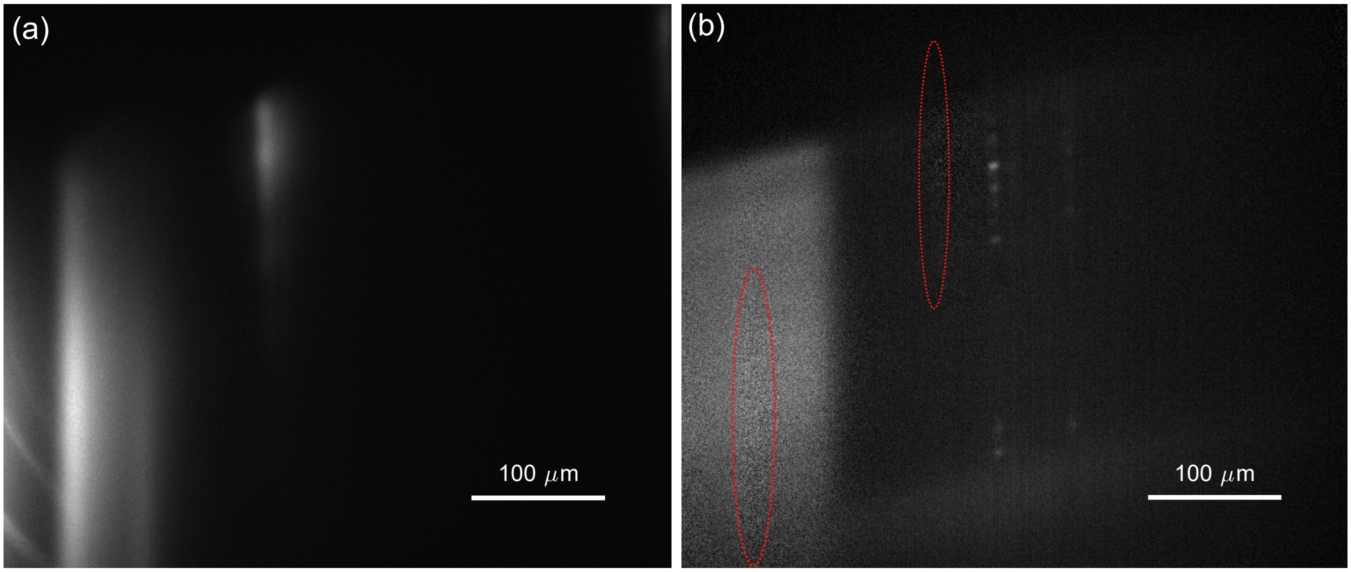

Fig. S3. Background scattering. (a) An image of the background scattering from intermediate optics measured with a beam block placed between the commercial microscope frame and the customized parts. (b) A hyperspectral image (with the beam block removed) showing artefacts (red circles) due to errors in background subtraction.

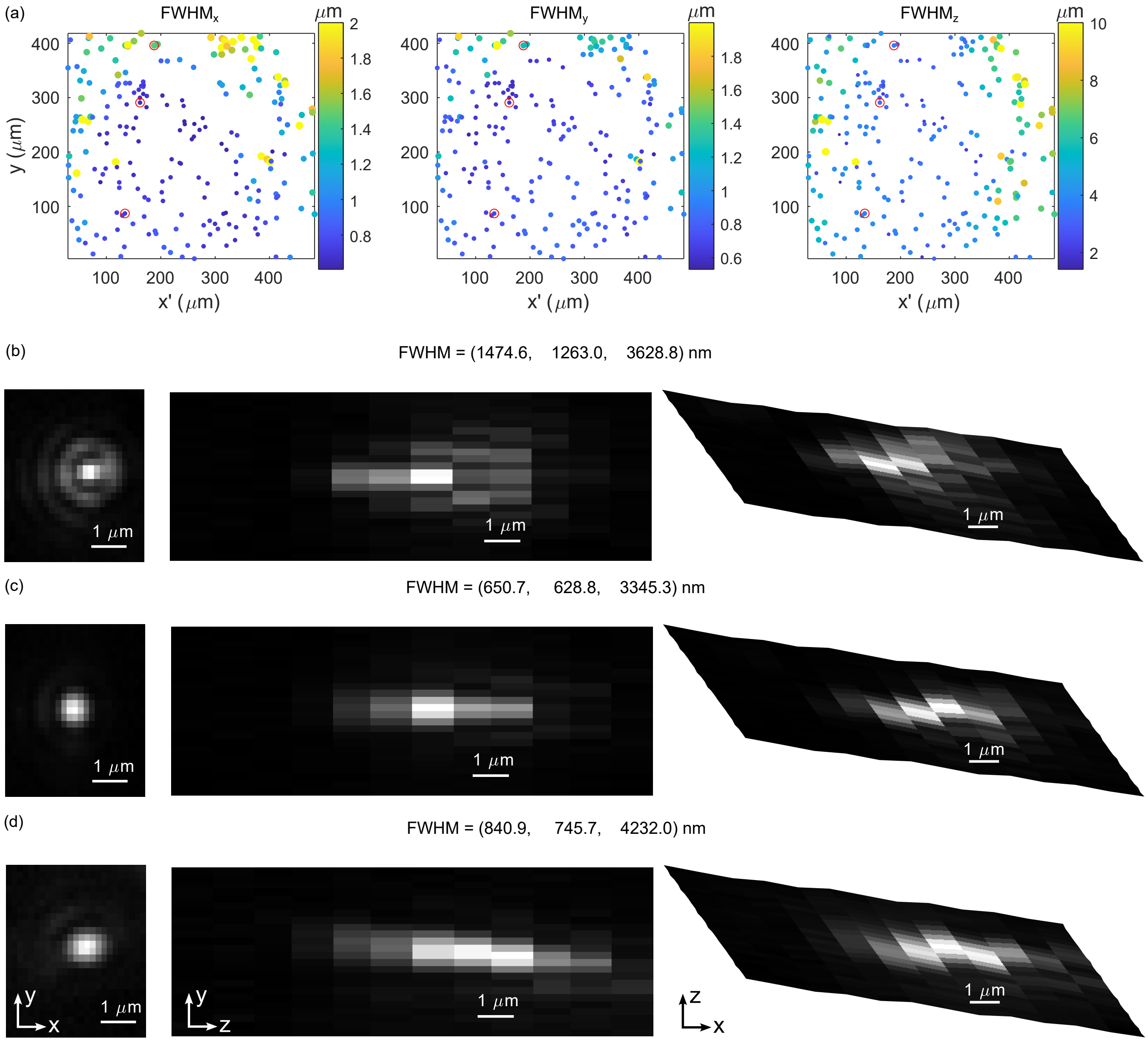

Fig. S4. Point spread function. (a) Distribution of the FWHM of the 3D PSF at different positions of the image. The resolution is evenly distributed in the middle $250\ \mu m \times 250\ \mu m$ area and increases at the edges of the image. The values are obtained by fitting each the 3D images of 224 fluorescent beads with 3D Gaussian functions (least square method). (b-d) Sections in the middle of the images of the 3 beads marked by red circles in (a) ordered from the top to the bottom of the field of view. The larger FWHM results from stronger aberrations near the edge of the field of view. Laser power: 5 mW, exposure time: 2 s.

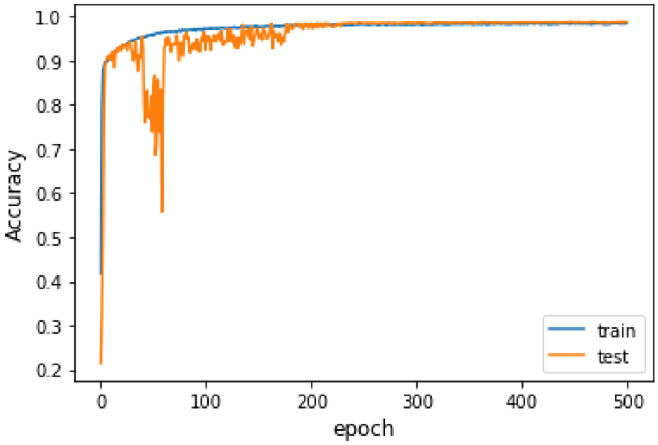

Fig. S5. Training and validation accuracy of the CNN model.

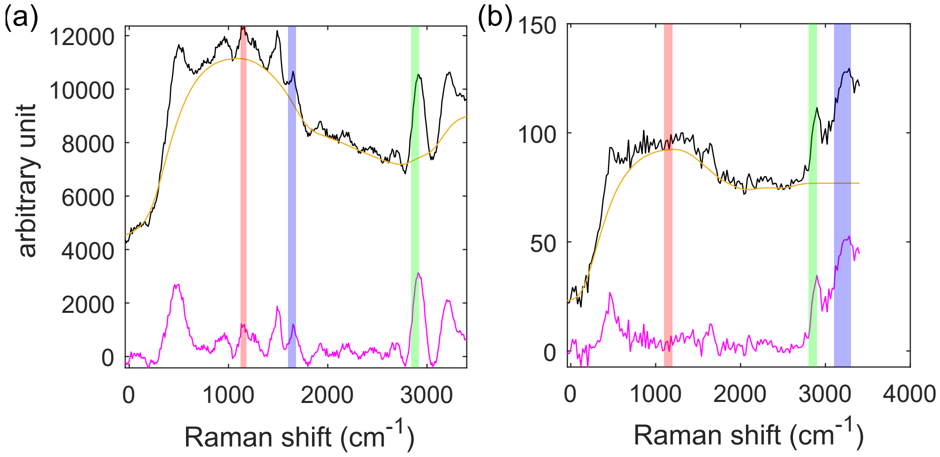

Fig. S6. Fluorescence subtraction. Example measured Raman spectra (black) and the corresponding estimated fluorescence spectra (orange) and the final spectra with fluorescence subtracted (magenta). (a) Spectra from the wound measurements. (b) Spectra from the heart measurement. The overlaying bars indicate the spectrum bands used for constructing the false colour maps in Fig. 3 and 4, with their colours indicating the corresponding RGB channels.

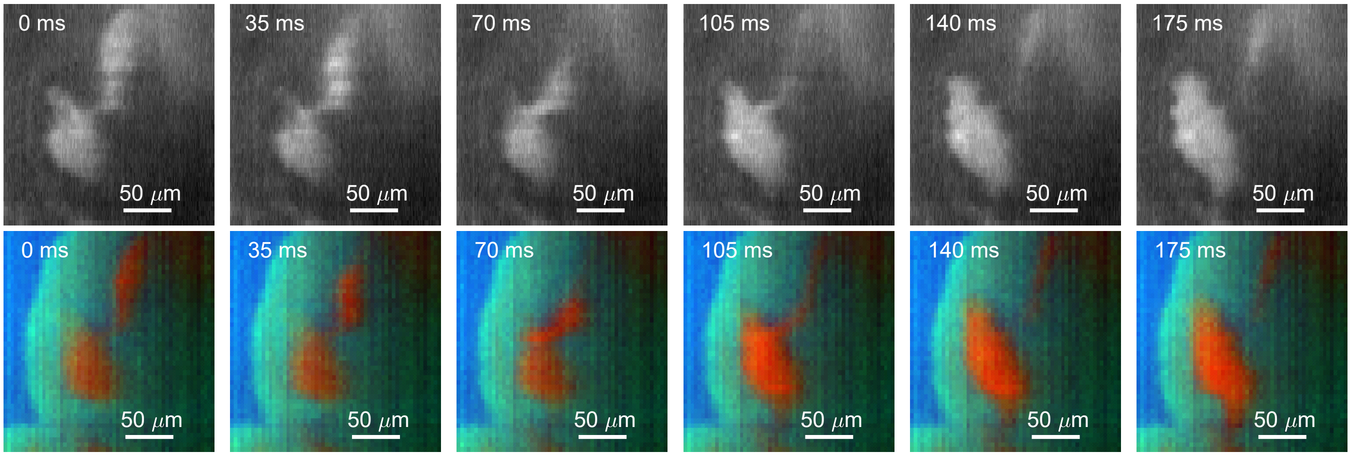

Fig. S7. Comparison of heart images from the two cameras. (Top): Raman images directly taken by Camera 1 at 6 time points, cropped and resized. (Bottom): corresponding processed Raman images obtained from hyperspectral images taken by Camera 2. The green, blue, and red channels indicate the distribution (normalized) of lipids/proteins (2800-2900 cm^{-1}), water (3100-3300 cm^{-1}) and auto-fluorescence (1100-1200 cm^{-1}) respectively. There is a slight difference between the horizontal positions of the two image sets.

Movie S1. A movie showing a $90 \times 52 \times 44$ 3D Raman scattering image of microplastics. The type of material at each position was identified using a convolutional neural network (CNN). Red: PS, green: PMMA, blue: polyamide / Nylon-6 (PA6), grey: agarose or dish. The image was binned and deskewed according to the tilt angle of the OPM and then rendered (with smoothening) using the 3D viewer of ImageJ. Original pixel size: $3.6\ \mu m \times 3.6\ \mu m \times 3.1\ \mu m$. The scalebar indicates 50 μm. Exposure time: 200 ms per line in x'. Total acquisition time: 7 min 42s, estimated laser power at the sample: 310 mW, $2.7\ mW/\mu m^2$.

Movie S2. A movie constructed from the images in Fig. 4 showing a beating heart with Raman contrast at the measured rate (28 fps).

References

1. B Yang, et al., Epi-illumination SPIM for volumetric imaging with high spatial-temporal resolution. *Nat Methods* **16**, 501–504 (2019).
2. E Sapoznik, et al., A versatile oblique plane microscope for large-scale and high-resolution imaging of subcellular dynamics. *eLife* **9**, e57681 (2020).
3. B Yang, et al., Daxi-high-resolution, large imaging volume and multi-view single-objective light-sheet microscopy. *Nat Methods* (2022).
